# Forest Loss in Protected Areas and Intact Forest Landscapes: A Global Analysis

**DOI:** 10.1371/journal.pone.0138918

**Published:** 2015-10-14

**Authors:** Matias Heino, Matti Kummu, Marika Makkonen, Mark Mulligan, Peter H. Verburg, Mika Jalava, Timo A. Räsänen

**Affiliations:** 1 Water and Development Research Group, Aalto University, Tietotie 1E, 02150, Espoo, Finland; 2 Natural Resources Institute Finland (Luke), Jokiniemenkuja 1, 01301, Vantaa, Finland; 3 Department of Geography, King's College London, Strand, London, WC2R 2LS, United Kingdom; 4 Environmental Geography Group, VU University Amsterdam, de Boelelaan 1087, 1081 HV, Amsterdam, the Netherlands; University of Guelph, CANADA

## Abstract

In spite of the high importance of forests, global forest loss has remained alarmingly high during the last decades. Forest loss at a global scale has been unveiled with increasingly finer spatial resolution, but the forest extent and loss in protected areas (PAs) and in large intact forest landscapes (IFLs) have not so far been systematically assessed. Moreover, the impact of protection on preserving the IFLs is not well understood. In this study we conducted a consistent assessment of the global forest loss in PAs and IFLs over the period 2000–2012. We used recently published global remote sensing based spatial forest cover change data, being a uniform and consistent dataset over space and time, together with global datasets on PAs’ and IFLs’ locations. Our analyses revealed that on a global scale 3% of the protected forest, 2.5% of the intact forest, and 1.5% of the protected intact forest were lost during the study period. These forest loss rates are relatively high compared to global total forest loss of 5% for the same time period. The variation in forest losses and in protection effect was large among geographical regions and countries. In some regions the loss in protected forests exceeded 5% (e.g. in Australia and Oceania, and North America) and the relative forest loss was higher inside protected areas than outside those areas (e.g. in Mongolia and parts of Africa, Central Asia, and Europe). At the same time, protection was found to prevent forest loss in several countries (e.g. in South America and Southeast Asia). Globally, high area-weighted forest loss rates of protected and intact forests were associated with high gross domestic product and in the case of protected forests also with high proportions of agricultural land. Our findings reinforce the need for improved understanding of the reasons for the high forest losses in PAs and IFLs and strategies to prevent further losses.

## Introduction

Forests play a crucial role in sustaining life on earth. They maintain ecological diversity, regulate climate, store carbon, protect soil and water and provide resources and livelihoods for the world’s population [1–4]. Despite the increasing awareness of the importance of these ecosystems, global deforestation rates have remained alarmingly high over the past decades [2].

In year 2010 forest covered around 40 million km^2^ or 31% of the global land area according to country reports [2]. Estimates of global forest loss rates within the past decade vary between 130,000 km^2^/yr [2] and 177,000 km^2^/yr [5]. At the same time, some reforestation and natural regrowth have occurred, leading to net forest loss reports ranging between 52,000 km^2^/yr and 115,000 km^2^/yr, respectively. Although forest loss is still remarkably high, it has shown some signs of decline: FAO [2] reports that during the 1990s, the forest loss rate was 160,000 km^2^/yr; whereas between 2000–2010, the rate was 130,000 km^2^/yr. According to FAO [2] the tropics were the only domain where the rate of forest loss increased in the first decade of the 21^st^ century compared to 1990s: deforestation increased in the tropics by 2,101 km^2^/yr on average across the decade. Deforestation in the tropics accounted for 32% of global forest loss within the period of 2000–2012 [5]. However, there are also positive signs in the tropics: the rate of forest loss in the Brazilian Amazon has declined in recent years [6,7].

The main drivers of global deforestation are linked to expansion of agriculture, wood extraction, infrastructure extension, population growth, and expansion of agriculture [8–13]. The dominant drivers, however, vary among the regions [8–13]. In addition to agriculture and population growth, a meta-analysis of 117 deforestation studies by Ferretti-Gallon and Busch [9] suggests that deforestation is generally lower in high, steep and wet areas while it is higher in areas where forests are closer to roads and urban areas. Deforestation has also shifted from a dominantly state initiated to an enterprise driven process in the tropics between 1970 and 2000 [14].

Alarming deforestation rates combined with the increasing awareness of the importance of forests have resulted in exponential growth of the world’s protected areas (PA) over the past decades [15–17]. Schmitt et al. [18] estimate the global forest cover using earth observation satellite data from MODIS2005 and the extent of protected forest using the World Database of Protected Areas (WDPA) for the year 2008. They find that 7.7% of global forest cover fell within IUCN’s four strictest protection categories (I-IV) and 13.5% within IUCN’s all six protection categories (I-VI) (IUCN protection categories vary from strictly protected areas (I) to protected areas with sustainable resource use (VI); for more detailed category definition see [19]). Schmitt et al. [18] further conclude that forest protection varied greatly between different regions and forest types, and that forest protection in priority areas, such as biodiversity hotspots, was insufficient.

Considerable efforts of protection are targeted to the primary forests and large intact forest landscapes (IFLs, i.e. unbroken expanse of natural ecosystems). These forest areas play crucial roles in sustaining ecological diversity [2,20]. FAO [2] estimates that primary forests accounted 14 million km^2^ (36% of the global total forests) in year 2010, having decreased alarming 400,000 km^2^ over the period of 2000–2010 (annual rate 0.4%). Potapov et al. [20] report that the extent of IFL is 13.1 million km^2^. Vast majority of the IFLs are found either in dense Tropical and Sub-Tropical forests (45% of world total) or in Boreal Forests (44%). The lowest proportion of IFL was found in Temperate Broadleaf and Mixed Forests. Potapov et al. [20] further find that 18.9% of IFLs are under protection of IUCN protection categories I-VI and only 9.7% of IFLs is strictly protected under IUCN protection categories I-III.

The protection effect of PAs is, however, questioned. The PAs are considered in many cases to be biased in their location, meaning that PAs are located in areas that are unlikely to face land conversion pressures [21]. Joppa and Pfaff [22] reveal that a majority of the PA networks are located in high elevations, steep slopes and far from roads and cities. Joppa and Pfaff [21] argue that this bias has resulted in overestimations in the protection effect of PAs. Local case studies (e.g. [23]) support these global findings. Joppa and Pfaff [24] use ‘matching’ approach that attempts to avoid the overestimation of protection effect of PAs in 147 countries by comparing protected and non-protected areas with similar land characteristics. They find that matching reduced the protection effect in 80% of the countries compared to an assessment without matching. Altogether they find that the protection reduced conversion of natural land cover in 75% of the assessed countries. Protected areas are also reported to become increasingly isolated, especially in tropics [25]. This is alarming because smaller protected areas are often under great threat [26] and isolation of these areas restricts the habitat size, i.e. limits the survival of a great number of fauna and flora species (e.g. [27,28]).Although the understanding of forest loss at a global scale with an increasing spatial resolution is growing rapidly [5], the global forest loss in PAs and in IFLs are not assessed with detailed and uniform datasets that allow consistent forest extent comparisons over space and time. So far only regional analyses exist at this level, for example, for Indonesia [29]. Furthermore, the success of protection in preserving the IFLs is not yet well understood. Therefore, in this study, we aim to conduct a consistent and spatially explicit assessment of the global forest loss, particularly within protected and intact forests by focusing on the variation among the countries between 2000 and 2012. Additionally, we aim to study whether socio-economic indicators can offer potential explanation to these observed global forest losses in PAs and IFLs. We hypothesised that i) the extent of forest loss within PAs and IFLs varies strongly among the countries but is always less manifested within PAs than outside of them, and ii) country level indicators of population size, land use change and state economy can offer potential explanation to the observed global forest losses in PAs and IFLs. The key terminology used in this study is explained in Table 1.

**Table 1:** Key terminology used in the study.

Term	Definition
Forest	Vegetation taller than 5 m with 20% tree cover canopy threshold using Global Forest Change (GFC) data by Hansen et al. [5].
Forest loss	”*Stand replacement disturbance or complete removal of forest canopy*” as defined by Hansen et al. [5] in GFC data. Forest gain was not taken into account.
Protected area (PA)	Protected area as in World Database on Protected Areas (WDPA) [30].
Protected forest	Forest within PA (i.e. protected area).
Intact forest landscape (IFL)	”*An unbroken expanse of natural ecosystems within areas of current forest extent*, *without signs of significant human activity*, *and having and area of at least 500 km* ^*2*^” as in IFL dataset by Potapov et al. [20].
Intact forest	Forest within IFL (i.e. Intact forest landscape).
Protected intact forest	Intact forest within PA (i.e. protected area).

## Materials and Methods

**Fig 1:**
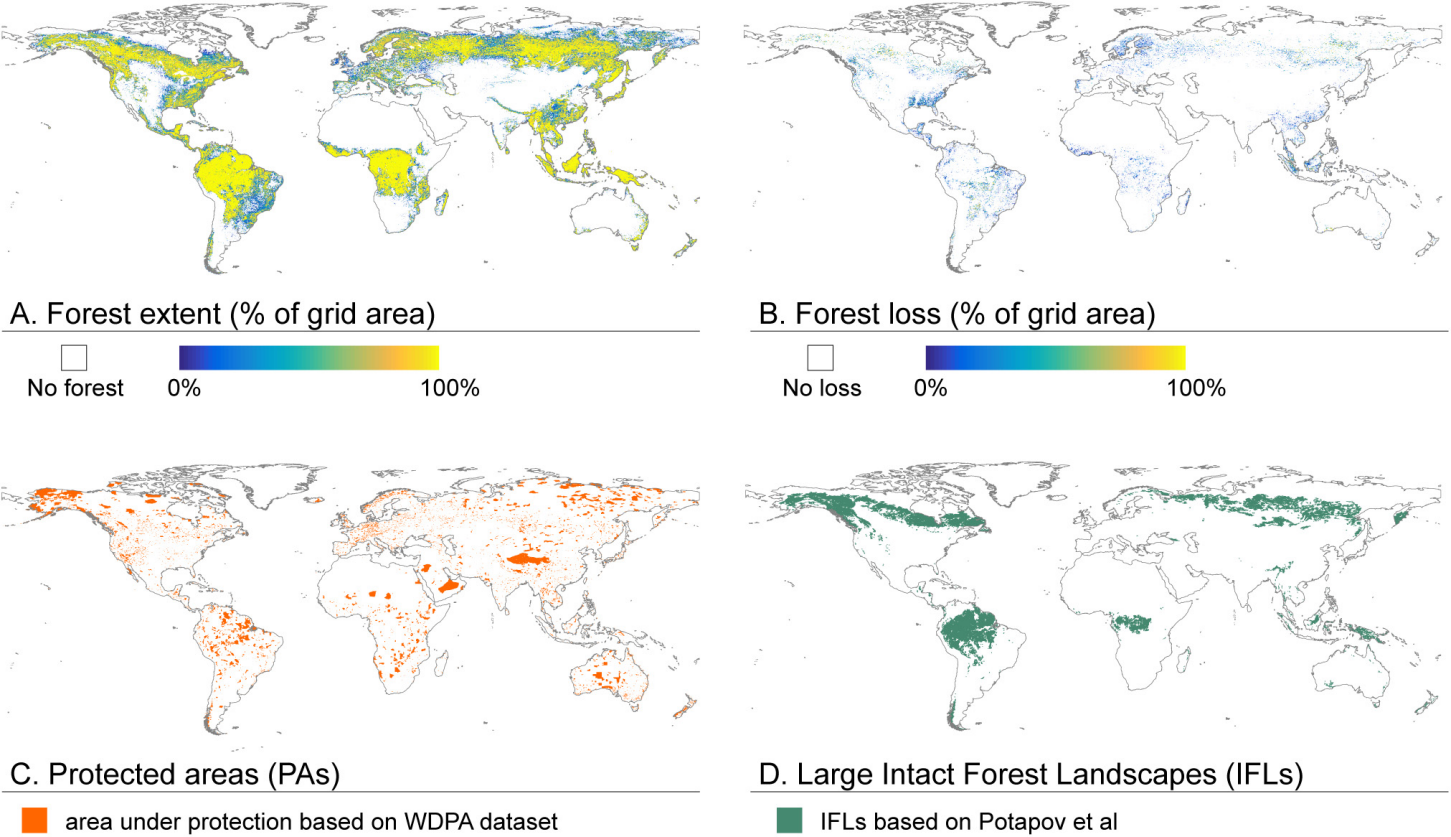
Aggregated (A-B) and converted (C-D) datasets used for the forest loss analyses. A) Forest extent in percentage of grid area at 1 km resolution, based on Global Forest Change (GFC) data [5]; B) Forest loss in percentage of grid area at 1 km resolution, based on GCF data [5]; C) Protected areas based on World Database of Protected Areas (WDPA) [30], converted to 1 km resolution; and D) Large Intact Forest Landscapes (IFLs) based on dataset by Potapov et al [20], converted to 1 km resolution.

To conduct the assessment, we combined four global datasets (Fig 1; Table 2): forest extent based on the Global Forest Change (GFC) data [5], forest loss based on the GFC data [5], the World Database of Protected Areas (WDPA) [30] and the global Large Intact Forest Landscapes (IFL) data [20]. From GFC we assessed the forest extent and forest loss while WDPA and IFL datasets were used to determine how much of that loss took place in protected areas and intact forest landscapes respectively. The resulting spatial data were aggregated to country-scale and analysed with Weighted Least-Squares (WLS) regression analyses to assess whether one or a series of socio-economic indicators are associated to forest loss patterns, possibly explaining their occurrence. The forest loss results are given at national and global scale and by geographical regions, while regression analyses were conducted at national scale to correspond with the socio-economic indicator data and the pursued forest governance level. Below the data and methods are described in more detail.

**Table 2:** A description of the datasets used in this study. GFC stands for Global Forest Change dataset.

Dataset	Spatial extent	Time period	Spatial resolution	Reference
GFC: Forest loss	Lat: 80N - 60S Lon: 90E - 90W	2000–2012	30 m (at equator; 1 arc-second x 1 arc-second)	Hansen et al. [5]
GFC: Forest extent	Lat: 80N - 60S Lon: 90E - 90W	2000	30 m (at equator; 1 arc-second x 1 arc-second)	Hansen et al. [5]
World Database of Protected Areas (WDPA)	Global	2010 (reference year)	Vector data with varying resolution	IUCN and UNEP-WCMC [30]
Large Intact Forest Landscapes (IFL)	Global	2000	Vector data with approximate resolution of 1 km (scale: 1:1,000,000)	Potapov et al. [20]

### Data

We used the GFC dataset [5] to map the forest extent and forest loss (Fig 1A and 1B; Table 2). GFC data are based on Earth observation satellite data and have a resolution of ~30 meters at the equator (i.e. 1 arc-second). The forest extent in the GFC data are given as canopy cover percentage per output grid cell for all vegetation taller than 5 meters for the year 2000 [5]. The forest loss in the GFC dataset is defined as stand-replacing disturbance, or complete removal of tree canopy, which has occurred between years 2000 and 2012 [5]. In this study, we used the same definition for forest loss (Table 1). The GFC dataset provides gross and net forest loss data. We chose to focus on gross forest loss instead of net forest loss, as we believe the former reflects better the occurrence of forest disturbances in protected and intact forests than the latter. The use of net forest loss could have masked some of the disturbances through forest gain.

It should be noted that the GFC dataset does not specify the reasons behind forest loss and thus, the reported forest loss inside protected areas might be due to direct human actions (e.g. illegal logging, management strategies) as well as natural causes (e.g. forest fires, diseases, pests and storms) [2,5,31,32]. For example, natural forest fires are reported to be the most significant cause of forest loss in the boreal forests [33].

We used the WDPA dataset to map the protected areas of the globe (Fig 1C; Table 2). The dataset is a joint project of IUCN and UNEP and it is stated to be the most extensive dataset on protected areas containing both nationally and internationally designated protected areas [30]. The dataset is based on both authority and community sources. We included only the protected areas reported by authority sources into our assessment.

For the intact forest areas we used the global extent of IFLs (Fig 1D; Table 2) [20]. The dataset is created using various remotely sensed datasets and maps, including data from MODIS and Landsat. They define IFL as an unbroken area of natural ecosystems within forests, without signs of significant human activity, and having an area of at least 500 km^2^. The reference year for this dataset is 2000, i.e. the starting year to our analysis.

### Methods: forest extent and forest loss

We conducted the analyses on 1 km resolution (at the equator; i.e. on 30 arc-second resolution). All datasets were either aggregated (forest extent and forest loss) or converted (PA and IFL) to this resolution. The conversion preserved the original resolution of the input data (Table 2) and the aggregation to coarser resolution was carried out in a way that original information was not distorted (see below for more details).

Before any data aggregation, we transformed the original GFC forest cover data into boolean type at 30 m resolution. For the transformation we used 20% tree canopy cover threshold, similarly to Potapov et al. [33], to define whether the 30 m resolution grid cells are classified as forest or no-forest. These boolean forest cover data were multiplied by a surface area of 30 m cell in question. The forest extent area data (in km^2^) were then aggregated into 1 km resolution by summing the surface area of forest from 30 m grid cells within each 1 km grid cell (note: the area of 30 m grid cells vary over latitudes in the original WGS84 projection and this was taken into account in area calculations). The GFC forest loss data are boolean data in nature (loss or no loss). These forest loss boolean data were multiplied by a surface area of 30 m cell in question and aggregated to 1 km resolution similarly as forest extent (see above). Possible forest loss was only accounted in those 30 m grid cells that had tree canopy cover over the 20% threshold in the forest extent dataset. As a result we got the forest extent and forest loss in km^2^ for each 1 km grid cell without losing any information in the aggregation process. To illustrate the aggregated datasets, the forest extent and forest loss in percentages of grid cell area are presented in Fig 1A and 1B.

IFL and WDPA datasets (Fig 1C and 1D; Table 2) were used to calculate the extent and loss of protected forest, intact forest and protected intact forest. We first converted the vector data of both datasets to raster data with 1 km resolution, being the original resolution of IFL data while resolution of WDPA data varies between the entries. After this we used the aggregated GFC data to calculate the area of forest extent and forest loss inside each type of spatial criteria within an analysis unit (country, region or global): i) total forest extent and loss, ii) forest extent and loss within protected areas, iii) forest extent and loss within intact forest landscapes, and iv) forest extent and loss within protected intact forest landscapes. We report both absolute and relative values for forest extent and loss.

By using these results, we analysed the forest protection effect of protected areas. The effect was estimated by calculating anomaly ratios for relative loss of protected and non-protected forest, intact and non-intact forest, and for protected intact and non-protected intact forest. The anomaly ratios were calculated at national scale by dividing the relative forest loss in the protected areas in question by the relative forest loss in the corresponding non-protected areas, e.g. [(relative loss of protected intact forest / relative loss of non-protected intact forest)– 1]. Thus the anomaly ratios provide information on the effect of protected areas on forest loss as they indicate whether the relative forest loss is lower (<0) or higher (>0) in the protected forest than in the non-protected forest.

We further identified similarities in forest loss patterns between countries by clustering the country scale results with *k*-mean clustering method. In this method the clustering of the observations (i.e. country-values) is based on minimizing the sum of the squared Euclidean distances between each observation and the cluster-centroid-value. We assessed goodness of fit in terms of variation within clusters. To define the optimal number of clusters we used the SSE curve. We performed two different cluster analysis, each considering four different parameters. In the first cluster analysis, we identified clusters based on absolute and relative losses in total forest and protected forest areas while in the second cluster analysis, we considered absolute and relative forest losses in intact forest landscapes and protected intact forest landscapes.

It should be noted that while for forest extent and intact forest datasets the reference year is 2000, for protected areas the coverage in year 2010 was taken. The reference year 2010 for protected areas was chosen because harmonization of the reference year was not feasible. The WDPA dataset does not report the establishment year for all protected areas. This however did not compromise the analyses as we found that the WDPA data from the year 2010 adequately reflect the protection status of the forest over the study period: only 8% of the worlds protected areas (where an establishment year is provided) were established after the year 2000.

### Methods: the relationship between forest loss and socio-economic indicators

To study the relationship between observed forest loss and socio-economic attributes, we employed WLS (i.e. Weighted Least Squares) regression analysis, which allowed the total extent of forest type in question to be used as weights. The degree to which the socio-economic attributes can account for the relative forest loss was analysed for each of the four forest loss measures considered in this study: total forest loss, loss in protected forest, loss in intact forest, and loss in protected intact forest. Altogether 11 commonly used socio-economic indicators were selected and they are listed in Table 3. The socio-economic indicators were, moreover, selected to represent the widely acknowledged causes for global deforestation: population size, land-use change, state governance and state economy. The selection was carried out with a requirement for available homogeneous data at the global scale. We briefly describe the WLS regression analysis below while step-by-step description is given in S3 Appendix.

**Table 3:** The socio-economic indicators used in the regression analysis. The indicators were used as independent variables in the assessment of the forest loss drivers with Weighted Least-Squares (WLS) regression analysis.

Variable	Data type	Time period	Reference
Population density	per square km	2010	UN [34]
Population density growth	%	2000–2010	UN [34]
GDP growth	%	2000–2010	World Bank [35]
GDP per capita (PPP)	International USD	2010	World Bank [35]
Rural population of total population	%	2010	World Bank [35]
Population growth	%	2000–2010	World Bank [35]
Corruption perception index ^a^	0.. 10	2010	Transparency International [36]
Polity index ^b^	-10.. 10	2010	Marshall and Gurr [37]
Human Development Index (HDI) ^c^	0.. 1	2012	UNDP [38]
Agriculture of total land area	%	2010	World Bank [35]
Agricultural land area growth	%	2000–2010	World Bank [35]

^*a*^ *Low corruption perception index corresponds with high corruption and vice versa*

^*b*^ *Low polity index corresponds with low democracy and vice versa*

^*c*^ *High HDI corresponds with high human development and vice versa*

The dependent variables were log_10_ –transformed prior to analyses to ensure the normality of their distributions. Similarly, the independent variables were transformed with a link function in cases it improved the linearity of the relation with the analysed forest loss measures. The link function of each independent variable was defined separately for each four dependent variables. Despite the transformations, the data included some statistical outliers. Exclusion of these outliers did not affect the significance of the results and thus the reported results were gained without omitting the statistical outliers. The multiple regression models were built by starting with the full model comprising of all 11 independent variables and ending with a model where no multicollinearity was detected. Multicollinearity was determined by the Variation Inflation Factor (VIF, a set limit to VIF < 4) and the collinear independent variable with the lowest explanatory power was always excluded from the model.

## Results

Our analyses based on GFC data [5] revealed that forest covered one third of the total land area (approximately 43 million km^2^) in the year 2000, and 19% of this forest (over 8 million km^2^) was under some form of protection (Table 4). According to our analyses 25% (11 million km^2^) of global forests were intact, of which almost 35% was protected. Areas that are both intact and protected covered thus a total of almost 3.7 million km^2^ (or 8.7% of the total forest extent) (Table 4).

**Table 4:** Forest extent in 2000 and its loss over the period of 2000–2012. The forest extent and loss are calculated for geographical regions in absolute [10^3^ km^2^] and relative values [%].

	Forest extent [10^3^ km^2^]				Forest loss [10^3^ km^2^]			
Region	Total extent	Protected extent (of total extent)	Intact extent (of total extent)	Protected intact extent (of total intact extent)	Total loss (of total extent)	Protected loss (of total protected extent)	Intact loss (of total intact extent)	Protected intact loss (of total protected intact extent)
Australia and Oceania	1,101	251 (23%)	256 (23%)	71 (28%)	48 (4%)	12 (5%)	4 (2%)	2 (3%)
Central America	1,038	221 (21%)	60 (6%)	47 (78%)	54 (5%)	11 (5%)	1 (1%)	0.4 (1%)
Eastern Asia	2,203	193 (9%)	32 (1%)	10 (33%)	70 (3%)	5 (3%)	1 (5%)	0.4 (4%)
Eastern Europe and Central Asia	8,759	1,067 (12%)	2,039 (23%)	300 (15%)	357 (4%)	43 (4%)	72 (4%)	10 (3%)
Latin America	9,665	3,322 (34%)	4,400 (46%)	2,415 (55%)	531 (5%)	41 (1%)	31 (1%)	9 (0.4%)
Middle and South Africa	6,768	1,054 (16%)	998 (15%)	212 (21%)	201 (3%)	22 (2%)	3 (0.3%)	0.4 (0.2%)
Middle East	188	12 (6%)	4 (2%)	0.4 (9%)	3 (2%)	0.1 (0.4%)	0.004 (0.1%)	0.0003 (0.1%)
North Africa	219	15 (7%)	- (-)	- (-)	2 (1%)	0.2 (1%)	- (-)	- (-)
North America	7,298	837 (11%)	2,408 (33%)	418 (17%)	513 (7%)	47 (6%)	152 (6%)	32 (8%)
South Asia	592	84 (14%)	31 (5%)	8 (26%)	10 (2%)	1 (1%)	0.1 (0%)	0.03 (0.3%)
Southeastern Asia	3,234	657 (20%)	478 (15%)	203 (42%)	271 (8%)	23 (4%)	3 (0.7%)	1 (0.3%)
Western Europe	1,481	353 (24%)	11 (0.8%)	9 (81%)	83 (6%)	12 (3%)	0.03 (0.2%)	0.01 (0.1%)
Global	42,562	8,068 (19%)	10,717 (25%)	3,693 (34%)	2,144 (5%)	219 (3%)	269 (2.5%)	55 (1.5%)

The global forest loss based on GFC data was 2.14 million km^2^ between the years 2000 and 2012, being over 5% of the total forest extent in year 2000 (Table 4). This global forest loss rate corresponds to calculations by Hansen et al. [5]. Further, according to our analyses, over 10% of the total forest loss occurred in protected areas: 219,000 km^2^ of protected forest (3% of the total protected forest) was lost within that time period. We further found that 269,000 km^2^ (2.5%) of intact and 55,000 km^2^ (1.5%) of protected intact forest were lost during the years 2000–2012 (Table 4). In the following sections the results are presented in more detail. The numeric results are also given for each country in S2 Table of the supplementary information. It is worth to highlight that our estimates on total forest extent and total forest loss on a global scale presented in this section are given only to validate our analyses (i.e. comparison to Hansen et al. [5]) and to put our results on PAs and IFLs into a context.

### Forest extents and their protection status

**Fig 2:**
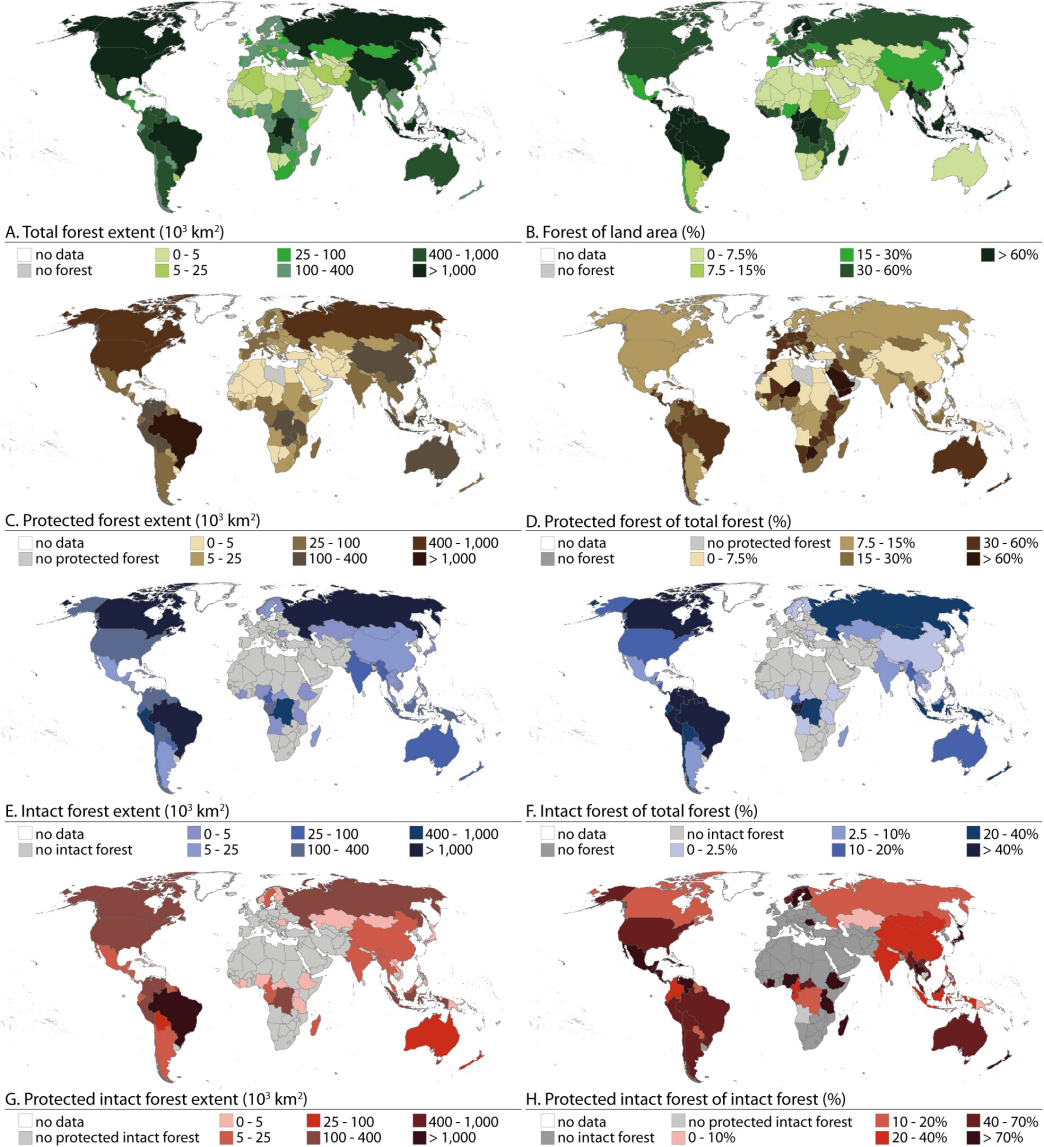
Absolute and relative forest extent by country in year 2000. Absolute forest extents (left column) are presented in km^2^ and relative forest extents (right column) are in %. A and B) Total forest extent; C and D) Protected forest extent; E and F) Intact forest extent; G and H) Protected intact forest extent.

In year 2000 the absolute forest extent was largest in Russia, China, Indonesia, Democratic Republic of the Congo, Brazil, United States, and Canada, exceeding 1 million km^2^ (Fig 2A). Together these countries contained over 60% of the global forest extent. When examining relative forest extent (Fig 2B), particularly Scandinavia and Finland (Nordic countries from here on), and countries in central Africa and Southeast Asia had a remarkable portion of their territory under forest. The countries with the largest protected forest areas were mainly the same countries with the greatest extent of forest (Fig 2C). However, when assessing the ratio of protected forest extent and the total forest extent, the largest protected forest areas were located in Central Europe, Australia, and in some countries of South America, Sub-Saharan Africa and the Middle East (Fig 2D), where over 30% of the forests were protected (though these often represent small areas of forest).

The countries with the largest total forest extent had also the largest areas of intact forest (over 400,000 km^2^), except Indonesia, United States and China (Fig 2E). On the other hand, a large part of European, Middle Eastern, North African and Southern African countries did not have any intact forest landscapes (Fig 1D). When comparing the area of intact forest to the area of total forest, we found that in many countries of North and South America, Southeast Asia, Middle Africa and Australia shares of intact forest of total forest were very high (Fig 2F). In contrast to these countries, Nordic countries had a large proportion of their land under forest (Fig 2B) but only less than 2% of that forest was classified as intact forest (Fig 2F).

Only Brazil had over 1 million km^2^ of protected intact forest (Fig 2G). Russia, Canada, United States and northern countries of South America had over 100,000 km^2^ of their intact forests under protection. When examining the relative extent of protected intact forest (extent of protected intact forest versus extent of total intact forest), we found that majority of intact forest, over 70%, were protected in countries such as Thailand, New Zealand, Japan, Madagascar, and Ethiopia as well as Nordic countries (Fig 2H). On the contrary, in some countries less than 20% of the intact forest was under protection, including Russia, Canada and Democratic Republic of the Congo (Fig 2H).

### Loss of total and protected forest

**Fig 3:**
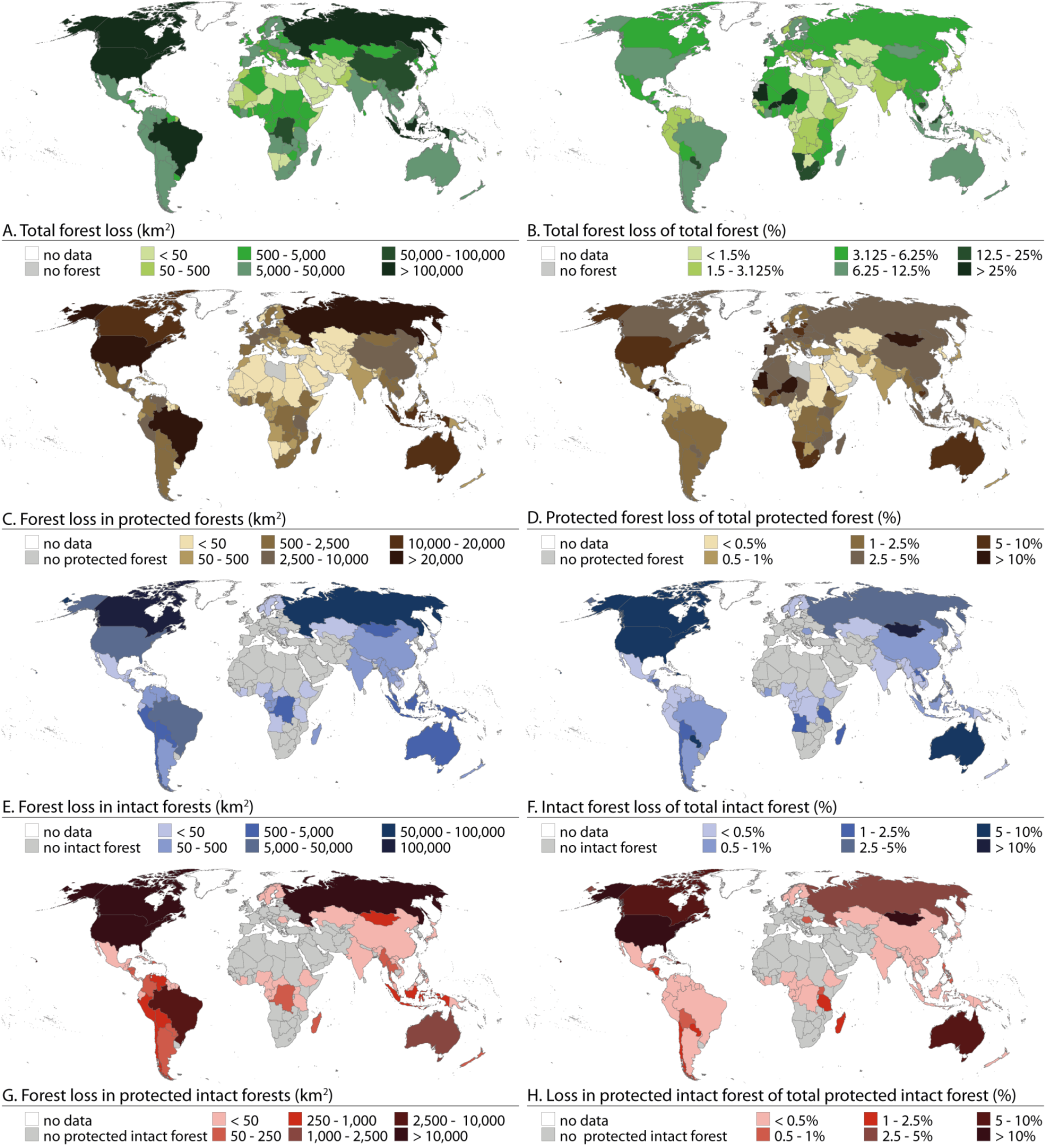
Absolute and relative forest losses by country between 2000 and 2012. Absolute forest losses (left column) are presented in km^2^ and relative forest losses (right column) are in %. A and B) Total forest loss; C and D) Loss in protected forests; E and F) Loss in intact forest; G and H) Loss in protected intact forests.

The largest absolute forest loss values were encountered in Brazil, Canada, United States, Russia and Indonesia. Each of them experienced forest losses of over 100,000 km^2^ during the period of 2000–2012 (Fig 3A). The largest relative losses, over 10% of their forest extent, were found in some countries in north-western and southern Africa, and Southeast Asia (Fig 3B). In these countries the relative losses were high in their protected forests too (Fig 3D). Relatively high (> 5%) protected forest losses occurred in Australia, United Stated and some European countries. South Asia, Middle East and Central Asia showed, on the other hand, small numbers of both absolute and relative protected forest losses (Fig 3C and 3D).

**Fig 4:**
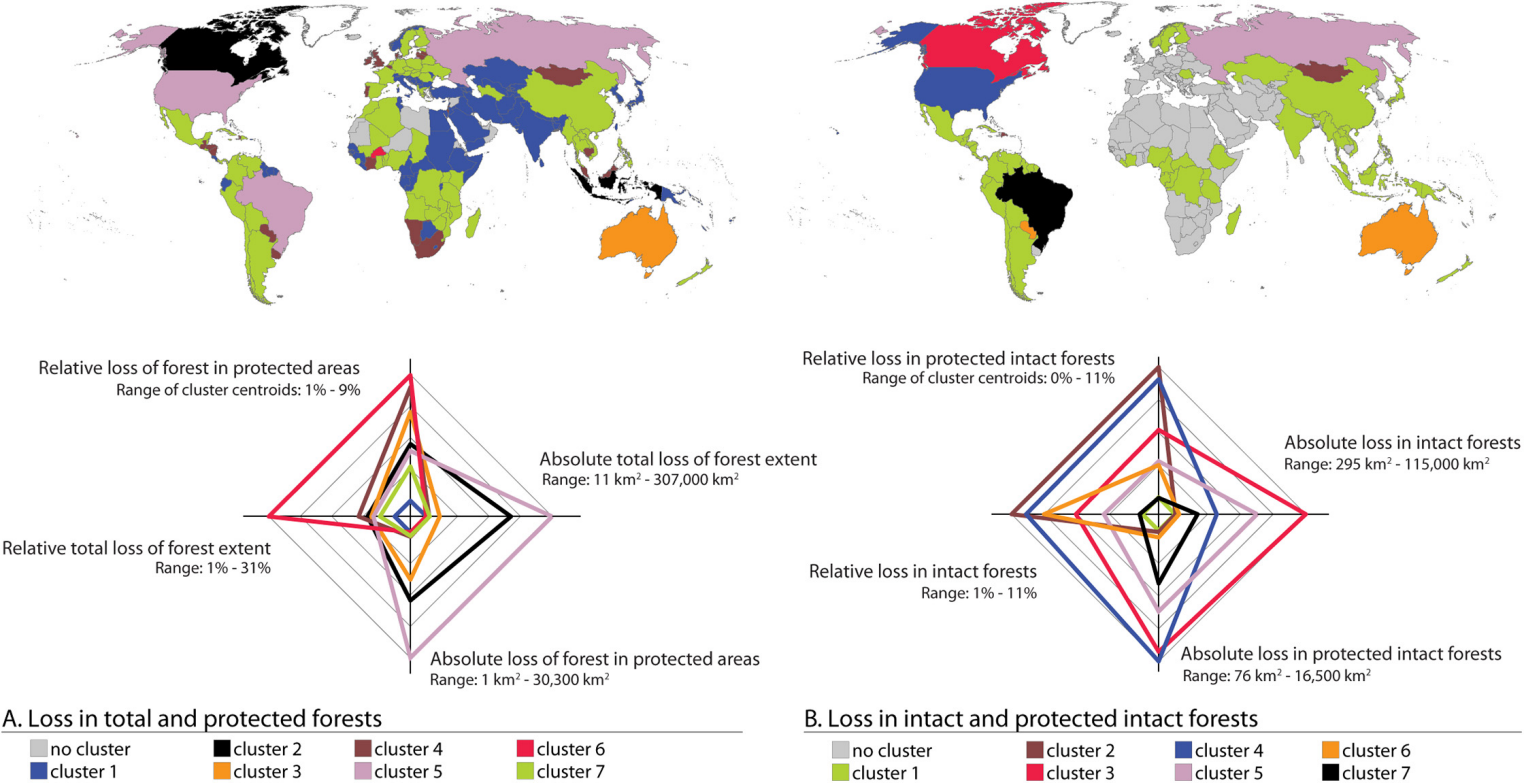
Clustering of countries by their forest loss patterns. A) country clusters based on absolute and relative losses in total and protected forest; and B) country clusters based on intact and protected intact forest. The cluster characteristics are illustrated by radar charts, of which radii represent the variables used in the clustering. The absolute range in each variable is given next to the variables. The cluster centroids on each variable are plotted on the radii, and these centroids are connected with lines. The countries within each cluster are mapped above the corresponding radar chart and the colours both, in the radar charts and their corresponding maps, indicate the cluster in question.

We were able to identify altogether seven distinct groups of countries differing by the nature of the total forest loss and loss in protected forest within the national territories (see Fig 4A). United States, Brazil and Russia formed a cluster with large areas of absolute loss in both total and protected forests while their relative losses were moderate (cluster #5 in Fig 4A; see also Fig 3A–3D). For the largest cluster with 100 countries, both absolute and relative forest losses were very small (cluster #1 in Fig 4A; see also Fig 3A–3D). The characteristics of another large cluster, consisting of 61 countries (cluster #7 in Fig 4A), were rather similar to the previous one, but the absolute and relative losses in both total and protected forests were slightly larger. One distinct cluster was formed also by Canada and Indonesia, together with some smaller islands in Southeast Asia, which all had moderate absolute and relative forest losses (cluster #2 in Fig 4A; see also Fig 3A–3D). The cluster with small absolute forest losses but moderately high relative losses includes countries such as Mongolia, South Africa and Paraguay (cluster #4 in Fig 4A; see also Fig 3A–3D).

### Loss of intact forest and protected intact forest

The largest losses of intact forest occurred in Canada with losses covering over 100,000 km^2^ (over 5% of the total intact forest), while Russia lost over 50,000 km^2^ of its intact forests and United States, Brazil and Australia over 5,000 km^2^ (Fig 3E). In relative terms, Northern America, Australia, and Mongolia lost the largest areas of their intact forest, as over 5% of their intact forests were lost compared to the intact forest extent in year 2000 (Fig 3F).

The largest absolute losses of protected intact forest occurred in Canada, United States and Russia, as all of them lost over 10,000 km^2^ of protected intact forests (Fig 3G). Large relative losses of protected intact forest (over 5% of total) occurred in Australia, Mongolia, Canada and United States, while smallest relative losses occurred in Asia, Nordic countries and South America (Fig 3H).

When considering similarities and differences between the losses in total intact and protected intact forest, we found that the countries with large areas (Brazil, United States, Canada and Russia) formed each a cluster of their own (see cluster results in Fig 4B). Each of them have very specific pattern in the loss results. For example in the case of Brazil, its absolute intact forest losses were relatively high, but the relative forest losses were rather small when compared to the other countries with the largest forest extents (Fig 3E–3H). Nordic countries, many countries in northern South America, central parts of Africa and South and Southeast Asia fell in the same cluster, having all very small absolute and relative losses in intact forests (Fig 3E–3H). Paraguay and Australia on the other hand, formed a cluster with the rather large relative losses of intact and protected intact forests while their absolute losses were rather small (Fig 3E–3H).

### The effect of protected areas

**Fig 5:**
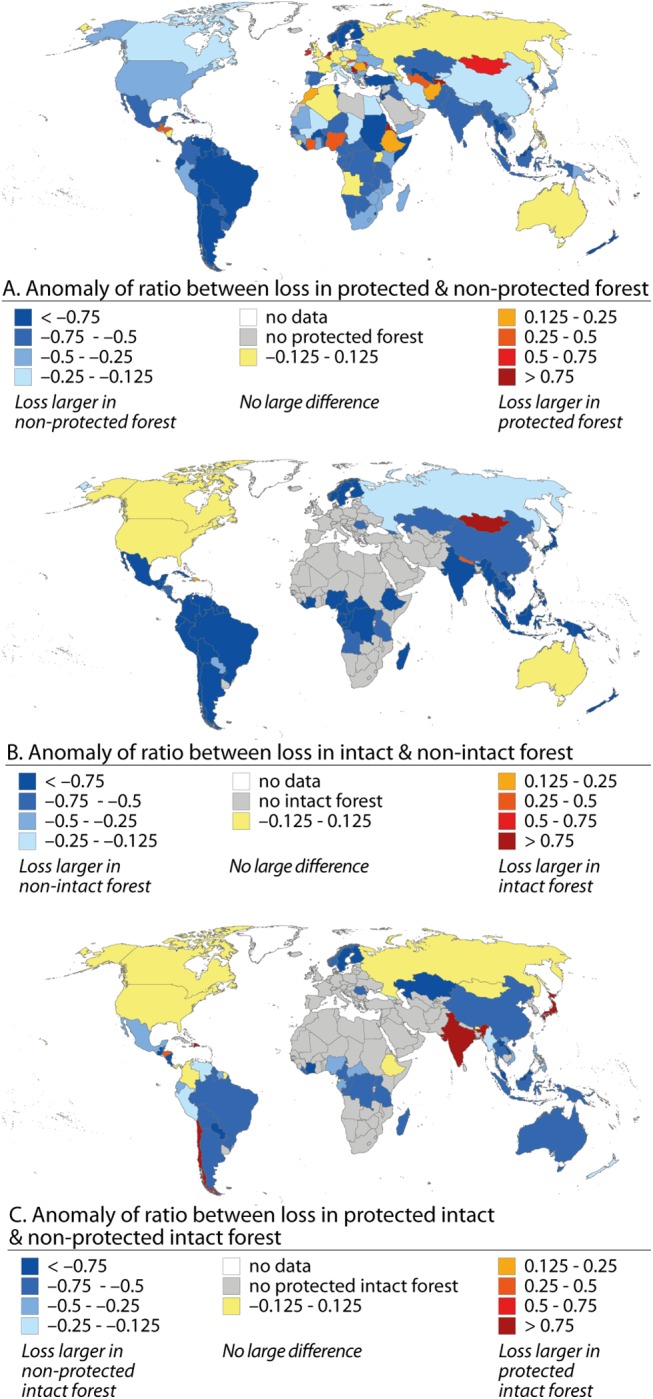
Comparison of forest loss between different forest types: A) Anomaly ratio for loss in protected forest and loss in non-protected forest; B) Anomaly ratio for loss in intact forest and loss in non-intact forest; C) Anomaly ratio for loss in protected intact forest and loss of non-protected intact forest (anomaly ratio <0 (>0) indicates that forest loss is lower (higher) in the protected forest type than in the non-protected forest type). Note: all the anomalies were calculated with relative losses.

Our findings revealed that the anomaly ratio for loss within protected forest and loss within non-protected forest was largely positive in some African countries, Netherlands, Afghanistan and Mongolia (Fig 5A), indicating that relatively more forest was lost in protected areas than in non-protected areas. The anomaly ratios were around zero in Russia, some African countries, Australia, and many countries in Europe. On the contrary, in a large number of countries in Latin America, Sub-Saharan Africa, Central and Southeast Asia, and Nordic countries, relative forest loss in protected areas was much smaller than in non-protected areas (Fig 5A).

When examining the anomaly ratios between intact and non-intact areas it can be seen that in Mongolia, Nepal and the Dominican Republic, it was more likely that the lost forest was intact than non-intact (Fig 5B). Australia, Canada, and United States experienced as much relative loss in intact forests as in non-intact forests. In Russia the loss in intact forest was close to the loss in non-intact forest (anomaly ratio is –0.2) while in the rest of the world’s countries with intact forest, the anomaly ratio was below –0.5. In these countries much less intact forest than non-intact forest (relative to total area of forest in both classes) was lost.

In a few countries, including India, Japan, and Chile, the relative losses in intact forest were much larger in protected areas than non-protected areas while in Canada, United States, Colombia, Mongolia, Ethiopia and Russia this anomaly ratio was around zero (Fig 5C) (i.e. relative loss in protected intact forest was equal to relative loss in non-protected intact forest). The smallest anomaly ratios (i.e. relative loss in intact forest is much smaller in protected than non-protected areas) were found in Nordic countries, large parts of Asia, Middle Africa, and Latin America (Fig 5C).

### The relationship between forest loss and socio-economic indicators

Each of the forest loss measures (total forest loss, loss in protected forest, loss in intact forest, and loss in protected intact forest), weighted by the forest area in question, had highly significant relationship with several socio-economic indicators (Table 5). All WLS regressions were highly significant and in the case of intact forest, the regressions explained over 75% of the weighted variations among countries in overall forest loss (Table 5).

**Table 5:** The associations of common socio-economic parameters to forest loss between 2000–2012. The four WLS regression models have each a different dependent variable: (a) relative total forest loss, (b) relative loss in protected forest, (c) relative loss in intact forest, and (d) relative loss in protected intact forest. The weight used in the regression for the forest loss measures was the total forest extent in question. Standardised beta coefficient (*B*) is given for each independent variable included in each of the four models. The independent variables are explained in Table 3. The country specific data are given in S1 Table and S2 Table of the supplementary.

	a) Forest loss	b) Protected forest loss	c) Intact forest loss	d) Protected intact forest loss
Independent variable	*B*	*B*	*B*	*B*
Population density	.028	–.170^#^	–.116	–.289***
Rural population	–.105	–.007	.073	.010
Population density growth	–0.001	.019	–.512***	–.441***
Population growth	–.022	–.165*	.021	.014
GDP per capita	.171	.450***	.606***	.600***
GDP growth	.131	–.015	-	-
Human Development Index (HDI)	-	-	-	-
Corruption Perception Index	-	-	-	-
Polity (i.e. level of democracy)	.457***	–.071	.190*	.136*
Agricultural land area of total land area	.245*	.254**	.086	.197**
Agricultural land area growth	–.032	–.028	.016	.209**
**WLS regression model**				
***n*** ^a)^	144	140	57	54
**r** ^**2**^	.367***	.314***	.781***	.862***

Statistical significance:

# *p* < 0.1

* *p* < 0.05

** *p* < 0.01

*** *p* < 0.001

^a)^ *n* varies over the models due to i) number of countries included in the model (e.g. not all countries have intact forest) and ii) depending on the socio-economic indicators included in the model, as indicators had some missing values.

Each of the four forest loss measures represents specific dimensions of forest loss during 2000–2012 and was best explained by a somewhat different set of socio-economic indicators. This variation could not be explained within the scope of the analyses, but it is expected that part of it originates from the different configuration of WLS regression models for different forest loss measures: the identity and number of countries varied between WLS regression models (Table 5). In addition, some of the used dependent variables were cross-correlated leading to a different choice of variables upon small differences between the dependent variables. The lack of socio-economic indicator data for all the countries also reduced the final number of countries included in the WLS regression models (see the *NaN* values for the socio-economic indicators in the S1 Table of the supplementary information).

Population density growth had, surprisingly, a negative relationship with weighted loss in intact and protected intact forests (Table 5). This indicates that an increase in population density is associated with a smaller forest loss. Population density itself had, similarly to population density growth, a negative relationship with weighted forest loss, although this relationship was only found with the loss in protected and protected intact forests. This indicates a stronger protection effect to prevent forest losses in densely populated countries. We found highly significant positive relationship between Gross Domestic Product (GDP) per capita and weighted loss in protected, intact, and protected intact forests: the higher the GDP, the higher the weighted forest loss. Moreover, polity (i.e. level of democracy) was also found to have a positive relationship with weighted forest loss (i.e. higher developed governance level was linked with higher forest loss) although, the statistical significance was observed in all forest loss types except in protected forest. Human Development Index (HDI) and corruption correlated highly with GDP (Tables A-D of S1 Appendix), and although they can as well offer explanatory power over forest loss, they had to be omitted from the final models.

More straightforwardly, agricultural land area had a significant positive relationship with total weighted forest loss and weighted loss in protected and protected intact forests. Similarly the agricultural land area growth had a positive relationship but only with weighted loss in protected intact forest (Table 5). In S2 Table of the supplementary information, we provide the country level data of our assessment that can be used for further analysis.

## Discussion

In our assessment of global forest loss between 2000 and 2012, the loss in protected and protected intact forests was found to be 3% and 1.5%, respectively, on global scale with a high variation among countries and geographical regions. The largest relative losses in protected forests occurred in North America (6%) as well as in Australia and Oceania (5%) and Central America (5%). When assessing the relative losses in intact forests, the largest regional losses were found in North America (6%) and in Eastern Asia (5%), where also protection did not prevent the high losses. Protection of forests and intact forests did not reduce forest loss in approximately one quarter of the analysed countries when the occurring loss within the protected areas was compared to outside of those areas. Looking at the relation between socio-economic indicators and global forest loss in PAs and IFLs, we found metrics of population density, GDP and agricultural land extent to provide probable causes for forest loss, although sometimes in a surprising direction.

### Forest extent and its loss

Our estimate for global forest extent based on data from Hansen et al. [5] show relatively consistent values when compared with previous studies (see a detailed comparison in S2 Appendix): our forest extent estimates are very close to those by FAO [2] and Schmitt et al. [18]. Our estimates, nevertheless, for global protected forest extent somewhat exceed the previous findings, as we included protected areas that are not assigned with IUCN protection category, while Schmitt et al. [18] focus on protected areas with IUCN protection category. When the same IUCN protection categories were used in the comparison, our findings would be very close to those by Schmitt et al. [18]. When focusing on the estimates of intact forests, the findings in this study indicate a smaller extent compared to the previous estimates by Potapov et al. [20] and FAO [2], who use primary forest extent as a somewhat equivalent measure for intact forests. When in turn focusing on the protected IFL extent estimates, our results indicate almost double the area compared to the findings by Potapov et al. [20]. But as above, the different definitions for protected areas by the two studies can cause the large difference (for more information, see S2 Appendix).

Looking at the forest loss, the annual average global forest loss estimates of FAO [2] are 34% smaller compared to this study and to the study by Hansen et al. [5], whose data we used. On contrary, while we found that 22,400 km^2^ intact forest was lost per year (Table 4), FAO [2] reports annual lost of 36,400 km^2^ of primary forest. Similarly to the comparison of forest extent, a direct comparison of forest loss between the present study and the study by FAO [2] is constrained by the different definitions used in the studies. Despite the problematic comparison between studies, the use of different definitions for assessed parameters related to global forest loss, expand the understanding of the issue.

The findings presented above thus reinforce the current knowledge of the global forest extent and loss concerning especially protected and intact forests. When focusing on these areas, we found that 19% of total forest extent and 34% of intact forest extent were within protected areas in year 2000. By year 2012 the extent of these protected forests had decreased by 3% and 1.5%, respectively (Table 4). These forest losses, furthermore, varied considerably between geographical regions supporting partly our first hypothesis. For example, loss in protected forest exceeded 5% in Australia and Oceania, and North and Central America, while the largest intact forest and protected intact forest losses were found in Eastern Asia and North America between 2000 and 2012 (Table 4; Fig 3B–3D).

### Effectiveness of protection

We used our analysis in protected areas to estimate the effectiveness of protection to prevent forest loss in those areas. Supporting further our first hypothesis, we found that in 146 of 191 assessed countries (76%), relative forest loss was smaller in protected areas than outside them (anomaly ratio < –0.125: Fig 5A). The protection, moreover, seemed to be particularly effective for reducing forest loss in South America, Southeast Asia and Sub-Saharan Africa (Fig 5A). Nevertheless, the effect of protection on forest loss was the opposite in some countries in Middle Africa, Europe, Middle East and Mongolia in Asia and thus rejects our first hypothesis of a unified positive protection effect. On country scale, our findings suggest positive news for Brazilian Amazon where protection was found to be very effective in preventing forest loss. This is in agreement with the earlier research findings on the Brazilian Amazon [6,39]. In the case of intact forest, the global results were similar, i.e. protection was effective in 43 of 60 of assessed countries (72%) (Fig 5B). Largest deviations to the global majority were shown by Mongolia, Nepal and Australia. It is worth to acknowledge that the PAs are not necessarily always aimed to protect the forest stands per se, instead protection can have other goals. Prior to our study, the effectiveness of protection is globally analysed by Joppa and Pfaff [24]. Our study further strengthens those findings, yet our findings are retrieved data-wise in more consistent manner by using temporally comparable forest extent datasets (Table 2), which only became available after the study by Joppa and Pfaff [24].

### Drivers of forest loss

Importantly, we also explored the drivers of forest loss by looking at the statistical linkage between socio-economic indicators and weighted forest loss (Table 5). Weighing the country specific forest losses by their total forest extent in question allowed our analysis to indicate the drivers that are expressive for the global forest extent instead of focusing on drivers explaining the inter-country variation. This globally uniform assessment showed forest loss having a strong connection with agricultural land extent. Moreover agricultural land expansion is recognised as one of the most important processes causing forest loss (see e.g. [8–13]) and our analysis agrees well with this, particularly concerning the protected intact forests (Table 5). However, it is worth to recognise that agriculture related forest loss involves much more complex dynamics than our regression analyses are able to capture (see e.g. [40]). We further found strong connection between losses in protected and/or intact forests and population density and GDP to fully support our second hypothesis.

While several global scale studies have found that higher population density increases forest loss (e.g. [41,42]), these all report total forest loss. We did not find significant relationship between total forest loss and population density, whereas our findings for losses in intact and protected intact forests (Table 5) show opposite direction compared to these existing studies on total forest loss. Our findings also seem to disagree with a meta-analysis by Porter-Bolland et al. [43]. However, a direct comparison between our results and those of Porter-Bolland et al. [43] is discouraged by the different scopes and approaches in the studies. Unlike our study, the meta-analysis assessed the effectiveness of only 40 protected areas in the tropics. Additionally, population densities are measured by different manners: in our study population densities were country averages while the meta-analysis based its data on population density in the immediate neighbourhood of protected areas. Our desire for single statistical analysis including multiple explanatory variables determined the selection of country averages on the population metric to match with the available level of the other variables. All in all, our findings together with those by Porter-Bolland et al. [43] point to a need for further research on the role of population dynamics in forest loss.

In terms of GDP, we did not find a statistically significant relationship with total forest loss (Table 5). This finding is in line with some previous studies [44–46], whereas some other studies suggest that lower GDP could indicate higher forest loss (e.g. [47,48]). While the effect of GDP on forest loss in protected areas has not been assessed previously on a global scale, it is evaluated by Nagendra [49] on 56 protected areas. Her findings indicate that the rates of land cover clearing do not differ significantly between countries with different levels of GDP per capita. We found, in turn, high GDP per capita to indicate high weighted forest loss rate in protected areas (Table 5). This relation between GDP and weighted forest loss on protected areas can possibly indicate different management strategies in countries with varying GDP, i.e. countries with higher GDP may have more versatile management strategies such as, those that aim to strengthen biodiversity or provide habitats for specific species, compared to countries with lower GDP.

Our assessment, i.e. looking at the forest loss drivers globally, naturally used rather limited set of socio-economic indicators on a coarse scale. This constriction on indicators was needed for the sake of homogeneous data. Another story would be revealed by more local and regional studies that could offer fruitful insights to this global study, as is for example shown by the global meta-analysis looking at the drivers behind the effectiveness of protection management [50]. Our study provides a global empirical assessment, weighted by the total forest extents in question, that can be used as a reference point for more detailed studies at other spatial scales.

### Taking further–subnational results and comparison among datasets

We acknowledge that the country level results presented in this study do not reveal finer level heterogeneity. Therefore, as an example, we explored how the forest loss in PAs varies within a country. Taking Colombia as an example, in our results we showed that the mean rate of forest loss within all protected areas was 0.08% per year. In a finer scale assessment using the same dataset (i.e. GFC) we found that the forest loss varied among the individual Colombian PAs from zero to 1.48%, the observed rates being low over large PAs in the Amazon and generally higher for protected areas in the Andes. The mean rate of annual forest loss per PA was 0.12% with standard deviation of 0.19%. This highlights the importance to study further the impact of protection on forest loss in lower spatial scales.

Furthermore, we carried out the same analysis by using an alternative dataset, Terra-I [51], to explore possible differences in available datasets. Terra-i dataset deviates from GFC in its use of MODIS (250 m resolution) instead of Landsat (30 m resolution) and 2004–2014 instead of 2000–2012. The range in forest loss in the individual protected areas (0% to 1.72%) was similar to the finding made by GFC but the mean annual forest loss rate within the PAs was one third (mean rate of 0.043% with standard deviation 0.14%) of the found losses compared to GFC dataset findings. This comparison reflects differences in data type, methodology and period but also indicates the uncertainty associated with these datasets and, thus calls for further comparisons between the widely used datasets.

### Methodological limitations and ways forward

It is important to expand the discussion towards the limitations created by the used datasets that also our study is naturally subjected to. For example, for the forest cover and loss we used the GFC dataset [5], which can give deviating results compared to other datasets mainly due to the differences in methods such as: definition of forest, separation of anthropogenic from naturally occurring forest loss, and handling of atmospheric distortions (e.g. cloud cover) in mapping the forest cover. Although, diving deeper into these differences and the effects they have on the findings is beyond the scope of this analysis, it would offer a fruitful topic for a comparative study. In addition, Lee [52] raises a concern on the IFL dataset [20] that its definition of intactness of forest may not capture the socio-ecological variation between different geographical regions and may thus introduce inaccuracies in country specific analyses. Inter-comparison of global scale datasets as well as more detailed analyses at the country/regional level would be needed to unravel the state of global classification of IFLs.

It should be noted that our study focused on forest loss, or more precisely on stand-replacing disturbance, ignoring the possible forest gain. This focus gave us more space to explore the loss of original habitats within protected areas and intact forest landscapes and the drivers behind these losses. A comparison of our findings to findings that would be based on the net forest loss, i.e. a loss assessment including the planting of forests and natural forest regeneration (see [2,5] and references therein for further information), would allow tracking of the forest canopy cover per se.

Our estimations on the effectiveness of protection to reduce forest loss within PAs compared to the forest loss outside those areas provide useful information the protection effect at the country level. At the country level, we were nevertheless not able to assess the leakage effect of individual protected areas, as done by e.g. Oliveira et al [53]. As highlighted by Ewers and Rodrigues [54] a comparison of forest loss in the vicinity of and inside protected areas is essential in understanding the effects of forest protection on a local scale. It is also worthwhile to recognise that forested areas might have been protected for other reasons than protect the forest stands per se. The global datasets used in this study could be further employed to this kind of assessment, comparing the leakage effect within and among regions and furthermore extending it all the way to global level. Most importantly, global data on forest management goals per each protection area would be highly needed to support more detailed assessment on the impact of protection on forest loss.

## Conclusions

Our global scale analysis indicates that substantial areas of protected (3%) and intact forests (2.5%) were lost over the past decade, and large part of that loss occurred in countries with well-established protection (e.g. in Australia and Oceania, and North America). In terms of comparison between forest loss inside and outside of PAs and IFLs we could not confirm our first hypothesis, which stated that forest loss would always be less manifested within PAs than outside of them. Although the relative forest loss was smaller inside the PAs and protected IFLs in global averages and in majority of countries, there were numerous countries that showed an opposite effect. Also our second hypothesis was not entirely confirmed: while population size and other indicators were able to explain differences in weighted global forest loss within PAs and IFLs, a large part of the variation remained unexplained. Moreover, the statistical associations did not always correspond with our causal understanding of forest loss processes. Our findings thus highlight that protection of forested areas does not always guarantee a lower rate of forest loss. There is, indeed, a high geographical variation in the effectiveness of protection against forest loss, a variation that is probably at least to some extent explained by countries’ different means and intensities to combat against forest loss.

## Supporting Information

S1 Appendix Cross-correlation tables. Cross-correlation tables of socio-economic indices in cases of different forest loss categories.
(PDF)

S2 Appendix Comparison of our findings to previous studies: forest extent and loss. (PDF)

S3 Appendix Weighted Least Squares (WLS) regression analysis steps. (PDF)

S1 Table Socio-economic data at country level. Socio-economic data used in the WLS regression analysis.
(XLSX)

S2 Table Country level results. Forest extent and forest loss results presented at country level.
(XLSX)
