# Supplementary material for: Forest Loss in Protected Areas and Intact Forest Landscapes: A Global Analysis
Source: PLoS One. 2015 Oct 14;10(10):e0138918. doi: 10.1371/journal.pone.0138918 (PMC4605629; doi:10.1371/journal.pone.0138918)
Supplement: S1 Appendix — Cross-correlation tables of socio-economic indices in cases of different forest loss categories. (PDF) [file pone.0138918.s001.pdf]

S1 Appendix: Cross-correlation tables

Table A. Cross-correlation table of socio-economic indices in case of total forest loss.

	pop density	pop density change	gdp change	gdp,ppp / cpt	rural pop	pop growth	corruption	polity	hdi	agric of land area	agric change
pop density		0.055	-0.254***	0.289***	-0.043	-0.001	-0.238**	0.138#	0.183*	-0.091	0.024
pop density change			0.065	0.002	-0.087	0.211**	-0.082	0.134#	0.117	0.102	0.021
gdp change				-0.131#	0.101	0.027	0.418***	-0.352***	-0.237**	0.141#	0.095
gdp,ppp / cpt					-0.574***	0.063	-0.634***	0.442***	0.833***	-0.394***	-0.044
rural pop						-0.142#	0.512***	-0.37***	-0.663***	0.19**	-0.039
pop growth							-0.153*	0.196*	0.201**	0.056	0.04
corruption								-0.627***	-0.731***	0.186*	0.033
polity									0.585***	0.041	-0.022
hdi										-0.203**	0.008
agric of land area											0.069
agric change											

Statistical significance:

#	$p < 0.1$
*	$p < 0.05$
**	$p < 0.01$
***	$p < 0.001$

Table B. Cross-correlation table of socio-economic indices in case of protected forest loss.

	pop density	pop density growth	gdp change	gdp,ppp / cpt	rural pop	pop growth	corruption	polity	hdi	agric of land area	agric land growth
pop density		-0.028	-0.117	-0.038	-0.035	-0.056	0.033	0.004	-0.037	-0.087	-0.032
pop density growth			-0.047	-0.102	-0.007	-0.032	0.053	-0.129	-0.155*	-0.181*	-0.006
gdp change				0.121	-0.017	-0.046	-0.174*	0.09	0.136#	-0.156*	-0.049
gdp,ppp / cpt					-0.079	0.085	-0.665***	0.483***	0.847***	-0.296***	-0.045
rural pop						-0.032	0.14#	-0.007	-0.116	0.095	-0.008
pop growth							-0.157*	0.19*	0.203**	0.043	0.039
corruption								-0.654***	-0.739***	0.166*	0.033
polity									0.605***	-0.004	-0.026
hdi										-0.197*	0.008
agric of land area											0.072
agric land growth											

Statistical significance:

#	$p < 0.1$
*	$p < 0.05$
**	$p < 0.01$
***	$p < 0.001$

Table C. Cross-correlation table of socio-economic indices in case of intact forest loss.

	pop density	pop density growth	gdp change	gdp,ppp / cpt	rural pop	pop growth	corruption	polity	hdi	agric of land area	agric land growth
pop density		0.011	-0.144	0.076	-0.183	-0.043	0.217#	0.167	0.185	-0.034	-0.104
pop density growth			0.092	0.069	-0.226#	-0.146	0.137	0.107	0.357**	0.013	-0.095
gdp change				0.215#	-0.162	-0.096	0.249#	0.301*	0.353**	-0.1	0.065
gdp,ppp / cpt					-0.337**	-0.082	0.607***	0.308*	0.687***	-0.293*	-0.09
rural pop						0.132	-0.359**	-0.325*	-0.643***	-0.062	0.322*
pop growth							-0.091	-0.195	-0.182	0.198	0.495***
corruption								0.445***	0.784***	-0.258*	-0.103
polity									0.501***	-0.101	-0.165
hdi										-0.119	-0.135
agric of land area											0.266*
agric land growth											

Statistical significance:

#	$p < 0.1$
*	$p < 0.05$
**	$p < 0.01$
***	$p < 0.001$

Table D. Cross-correlation table of socio-economic indices in case of protected intact forest loss.

	pop density	pop density growth	gdp change	gdp,ppp / cpt	rural pop	pop growth	corruption	polity	hdi	agric of land area	agric land growth
pop density		0.005	0.224#	0.068	-0.005	-0.079	0.191	0.162	0.172	-0.036	-0.397**
pop density growth			0.071	0.06	-0.088	-0.113	0.143	0.069	0.345**	0.021	-0.266*
gdp change				-0.214	0.005	-0.007	-0.309*	-0.383**	-0.312*	0.152	-0.109
gdp,ppp / cpt					-0.07	-0.068	0.626***	0.253#	0.685***	-0.292*	-0.139
rural pop						0.047	-0.088	0.003	-0.157	-0.165	0.032
pop growth							-0.075	-0.102	-0.157	0.226#	0.19
corruption								0.418**	0.811***	-0.253#	-0.406**
polity									0.43***	-0.095	-0.285*
hdi										-0.114	-0.386**
agric of land area											-0.082
agric land growth											

Statistical significance:

#	$p < 0.1$
*	$p < 0.05$
**	$p < 0.01$
***	$p < 0.001$
