# Supplementary material for: Forest Loss in Protected Areas and Intact Forest Landscapes: A Global Analysis
Source: PLoS One. 2015 Oct 14;10(10):e0138918. doi: 10.1371/journal.pone.0138918 (PMC4605629; doi:10.1371/journal.pone.0138918)
Supplement: S2 Appendix — (PDF) [file pone.0138918.s002.pdf]

S2 Appendix: Comparison of our findings to previous studies: forest extent and loss

Comparison of our findings to the findings of earlier studies reveals that all studies show relatively consistent values for global forest extent, deforestation and protection status of forests. The global forest extent was estimated to be 40.9 million (year 2000) and 39 million km^2 (year 2005) by FAO [1] and Schmitt et al. [2], respectively. This corresponds rather well to our estimate of 42.6 million km^2 for the year 2000. The estimate of FAO [1] is based on country reports while the estimate of Schmitt et al. [2] is based updated Global Forest Map (GFM) with 10% tree cover threshold. Our estimate is based on the GFC data from Hansen et al. [3] with 20% canopy cover threshold (Table 4).

Around 13% of the global forest is under protection according to FAO [1] and Schmitt et al. [2]. Our results, indicate a somewhat larger share of the world's forests are under some form of protection, reaching 19% (Table 4). The protection estimate of FAO [1] is based on country reports and the estimate of Schmitt et al. [2] is based on updated GFM data, WDPA data on IUCN protection category I-VI protected areas from the year 2008 with a 10% tree cover threshold. Our results are rather close to those in the previous literature, if only the IUCN protection category I-VI areas are taken into account as this resulted in protection share of 11.5% of the global forest extent. If only the IUCN protection categories I-IV are included, our results indicate 7.0% protection, while Schmitt et al. [2] report a share of 7.7%.

The global extent of IFLs (i.e. intact forest landscapes) and primary forests has been estimated by Potapov et al. [4] and FAO [1], respectively. Potapov et al. [4] report the extent of IFL to be 13.1 million km^2 in the year 2000 while FAO [1] report that the extent of primary forest at 14.7 million km^2. Using the Potapov et al. [4] IFL areas in combination with the Hansen et al. [3] forest extent, we estimated the intact forest extent in the year 2000 to be somewhat smaller than reported elsewhere, 10.7 million km^2 (Table 4). Forest cover estimate of Potapov et al. [4] is based on tree cover canopy dataset from MODIS 2005 (resolution of 500 m) and 20% canopy cover threshold.

Potapov et al. [4] report that 18.9% of the intact forest is protected whereas our estimate was 34% (Table 4). Both studies, our and Potapov et al. [4], used the same dataset for the definition of intact forest landscape. The difference may originate from the different estimates of the extent of intact forest (see above) and from the different selection of categories of protection status. Potapov et al. [4] included only the IUCN protection categories I-VI from the WDPA data, whereas our study included more liberally all authority reported protected areas (also including IUCN protection categories I-VI) from the WDPA data for the year 2010.

The global forest loss was estimated by FAO [1] to be 1.43 million km^2 over the period 2000-2010 while Hansen et al. [3] reported that over the period 2000-2012 around 2.3 million km^2 forest was lost (gross loss). Our assessment, based on data from Hansen et al. [3] and using 20% canopy cover threshold, revealed that forest loss rate over the period 2000-2012 was 2.1 million km^2. The forest loss estimates of FAO [1] are based on country reports, while the estimates of Hansen et al. [3], and thus ours, are based on Landsat data with 30 m resolution.

FAO [1] further reported that during the period 2000-2010 approximately 400,000 km^2 of primary forest was lost whereas our assessment reveals that 269,000 km^2 of intact forest area was lost during the period 2000-2012 (Table 4). FAO [1] used country reports for the primary forest area estimates where the definition of primary forest might differ from country

to country, whereas we used globally coherent data [3,4]. Therefore, our results provide more comparable results among different countries.

References

1. FAO (2010) Global forest resource assessment 2010. Rome: Food and Agriculture Organisation of the United Nations.

2. Schmitt CB, Burgess ND, Coad L, Belokurov A, Besançon C, et al. (2009) Global analysis of the protection status of the world's forests. Biological Conservation 142: 2122-2130.

3. Hansen MC, Potapov PV, Moore R, Hancher M, Turubanova SA, et al. (2013) High-resolution global maps of the 21st-century forest cover change. Science 342: 850-853.

4. Potapov P, Yaroshenko A, Turubanova S, al. e (2008) Mapping the world's intact forest landscapes by remote sensing. Ecology and Society 13.
