# Supplementary material for: Forest Loss in Protected Areas and Intact Forest Landscapes: A Global Analysis
Source: PLoS One. 2015 Oct 14;10(10):e0138918. doi: 10.1371/journal.pone.0138918 (PMC4605629; doi:10.1371/journal.pone.0138918)
Supplement: S3 Appendix — (PDF) [file pone.0138918.s003.pdf]

# *S3 Appendix: Weighted Least Squares (WLS) regression analysis steps*

Altogether four parallel WLS regression analyses were performed with a uniform procedure. The four regression analyses differed only by the dependent variable. The dependent variables were forest loss in four categories (i.e. total forest loss, loss in protected forest, loss in intact forest, and loss in protected intact forest). The uniform regression procedure is presented below step by step, to support the description given in Section 2.3 of the main article.

1. **Transformation of dependent variable** (i.e. forest loss percentage in question): dependent variables were $\log_{10}$ –transformed prior to analyses to ensure the normality of their distributions. We tested the normality with Shapiro-Wilk test with significance threshold of 0.05 for alpha.

2. **Transformation of independent variables** (i.e. socio-economic factors): independent variables were transformed with a link function in case the link function could improve the linearity of the relation with the analysed forest loss measures. The link function for each independent variable was defined separately for each four dependent variables. We tested altogether 10 different link functions, of which we selected the best one by comparing the $R^2$ of correlation between $\log_{10}$-transformed dependent variable and independent variable in question.

3. **Cross-correlation of independent variables:** to support the WLS regression analysis, we run cross-correlations between all independent variables. This is provided in S1 Appendix.

4. **WLS regression:**

    a. For each forest loss category we created an input dataset using the transformed dependent and independent variables. The countries with NaN values in dependent variable were excluded in this stage.

    b. We used SPSS v23 to conduct the WLS regression analysis

    c. The multiple regression model was built by starting with the full model comprising of all 11 independent variables and ending with a model where no multicollinearity was detected. Multicollinearity was determined by the Variation Inflation Factor (VIF, a set limit to VIF < 4) and the collinear independent variable with the lowest explanatory power was always excluded from the model.

    d. Once we found a model with no multicollinearity, we checked the model for possible outliers in the data. The possible outliers were detected visually from a biplot of standardized residuals and standardized predicted values. In case we detected outliers, the model was rerun without the outliers and the model outcome was compared to the model outcome with the outliers included in the data.
